# Gemcitabine, a broad-spectrum antiviral drug, suppresses enterovirus infections through innate immunity induced by the inhibition of pyrimidine biosynthesis and nucleotide depletion

**DOI:** 10.18632/oncotarget.23258

**Published:** 2017-12-15

**Authors:** Kyungjin Lee, Dong-Eun Kim, Kyoung-Soon Jang, Seong-Jun Kim, Sungchan Cho, Chonsaeng Kim

**Affiliations:** ^1^ Center for Convergent Research of Emerging Virus Infection, Korea Research Institute of Chemical Technology, Daejeon, South Korea; ^2^ Department of Medicinal and Pharmaceutical Chemistry, Korea University of Science and Technology, Daejeon, South Korea; ^3^ Anticancer Agent Research Center, Korea Research Institute of Bioscience & Biotechnology, Cheongju, South Korea; ^4^ Department of Biomolecular Science, KRIBB School of Bioscience, Korea University of Science and Technology, Daejeon, South Korea; ^5^ Biomedical Omics Group, Korea Basic Science Institute, Cheongju, South Korea

**Keywords:** enterovirus, gemcitabine, antiviral drug, pyrimidine biosynthesis, interferon-stimulated genes (ISGs)

## Abstract

Gemcitabine, an anti-cancer chemotherapy drug, has additionally shown the antiviral activity against a broad range of viruses and we also have previously reported its synergistic antiviral activity with ribavirin against enteroviruses. As a cytidine analog, gemcitabine has been reported to have an inhibitory activity on the pyrimidine biosynthesis. In addition, a few inhibitors of the pyrimidine biosynthesis have shown to induce the innate immunity in a yet-to-be-determined manner and inhibit the virus infection. Thus, we also investigated whether the anti-enteroviral activity of gemcitabine is mediated by innate immunity, induction of which is related with the inhibition of the pyrimidine synthesis. In this study, we found that the addition of exogenous cytidine, uridine and uridine mono-phosphate (UMP) effectively reversed the antiviral activity of gemcitabine in enterovirus-infected as well as enteroviral replicon-harboring cells, demonstrating gemcitabine’s targeting of the salvage pathway. Moreover, the expression of several interferon (IFN)-stimulated genes (ISGs) was significantly induced by the treatment of gemcitabine, which was also suppressed by the co-treatment with cytidine. These results suggest that the antiviral activity of gemcitabine involves ISGs induced by the inhibition of the pyrimidine biosynthesis.

## INTRODUCTION

Enteroviruses belong to the *Picornaviridae* family, which is characterized by a single stranded positive-sense RNA genome with about 7500-8000 nucleotides, and have been emerged as the major causative agents of various human diseases. Coxsackievirus B3 (CVB3), one of the most well-studied enteroviruses, causes viral meningitis, myocarditis and pancreatitis [1, 2]. In addition, enterovirus 71 (EV71) is a causative agent of hand-foot-mouth disease and also of severe neurological symptoms, which can lead to even death [3–5]. However, despite the increasing public threat, no effective therapy is currently available for the treatment of these infections.

Enteroviruses have hundreds of distinct viruses, and newly emerging enteroviruses have been increasingly reported in recent years. Moreover, many RNA viruses including influenza, severe acute respiratory syndrome coronavirus (SARS-CoV), Middle East respiratory syndrome coronavirus (MERS-CoV) and zika virus (ZIKV) have become an enormous threat for public health. Therefore, broad-spectrum antiviral drugs are necessary to efficiently control various viral infections. In another aspect ineffectiveness of conventional enzyme-targeting drugs due to the rapid development of resistant mutants is another hurdle we need to tackle. In order to achieve the development of broad-spectrum antiviral drug with a low rate of mutation, two strategies have been generally considered. One is targeting host cellular factor that is essentially required for the viral life cycle. This strategy would have a low potential of producing resistant viruses, but undesirable side effects could be accompanied. The other is activating innate immune response such as interferon (IFN) signaling so as to boost host antiviral defense system [6–9]. Actually, IFN itself or in combination with other antiviral drugs such as ribavirin has been primarily used for the treatment of various RNA virus infections. More recently, a few inhibitors of nucleoside biosynthesis have been shown to induce the innate immunity and suppress a broad range of virus infections [10–14]. For instance, Wang et al identified a broad-spectrum antiviral compound (Brequinar) targeting DHODH, a key enzyme of the pyrimidine biosynthetic pathway, and subsequently inducing innate immune response [10].

Previously, we identified gemcitabine, a drug currently being used for anti-cancer chemotherapy, as an effective inhibitor of enteroviruses including CVB3, EV71 and human rhinoviruses (HRVs) [15]. Its antiviral activity has been also shown against various RNA viruses including hepatitis C virus (HCV), human immunodeficiency virus (HIV), influenza virus, poliovirus, MERS-CoV and ZIKV [16–21]. Gemcitabine, as a cytidine analog, was reported to interfere with the pyrimidine biosynthesis [22]. However, the role of pyrimidine inhibition and the involvement of subsequent innate immunity in the antiviral action of gemcitabine have not been explored yet.

In this study, we examined the role of pyrimidine inhibition in the antiviral activity of gemcitabine by adding the exogenous nucleosides to CVB3-infected or CVB3 replicon-harboring HeLa cells. As a result, the antiviral effect of gemcitabine was remarkably suppressed by the pyrimidine nucleosides. Further analysis demonstrated that gemcitabine inhibited the salvage pathway of pyrimidine biosynthetic pathway most probably by targeting cytidine and/or uridine synthesis. Moreover, the treatment with gemcitabine activated the expression of several IFN-stimulated genes (ISGs), the major effectors in the innate immunity, which was also suppressed by the supplemented cytidine.

## RESULTS

### Suppression of the antiviral activity of gemcitabine by exogenous pyrimidine nucleosides

**Figure 1:**
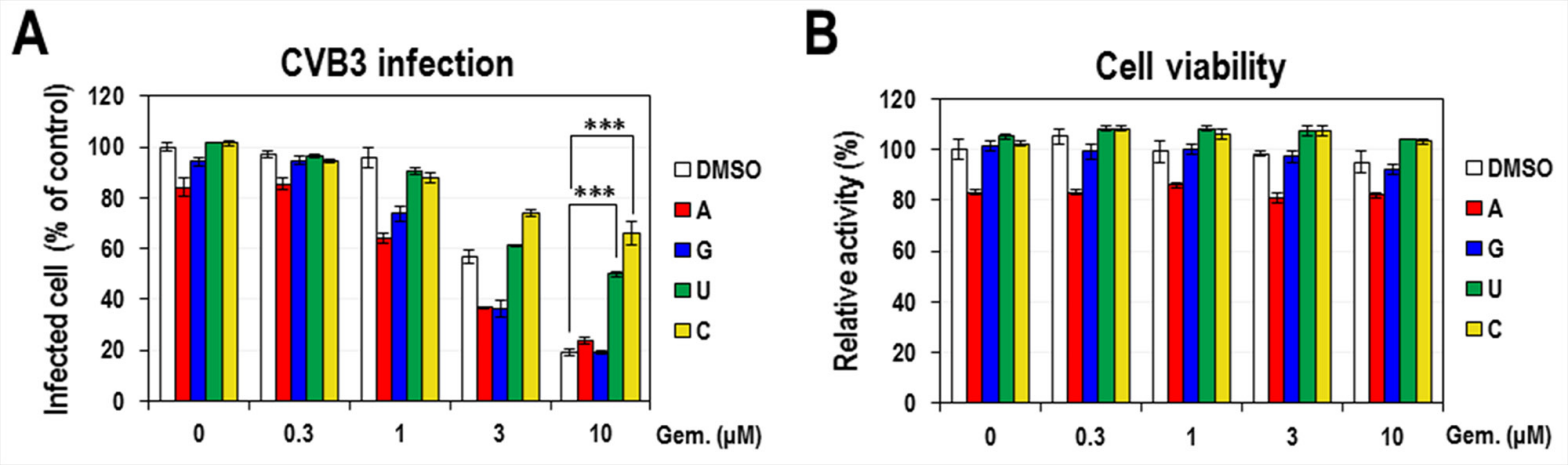
The effect of exogenous nucleosides on the anti-CVB3 activity of gemcitabine **(A)** HeLa cells were infected with CVB3 and simultaneously treated with various concentrations of gemcitabine and 100 μM of 4 nucleosides. At 8 hours post-infection the virus-infected cells were visualized by staining with anti-CVB3 3Cpro antibody, and the percentage of infected cells among total cells was calculated by setting the value from DMSO-treated cells as 100%. The average and standard deviation were obtained from three independent experiments. ^***^ indicates *P* < 0.001. **(B)** HeLa cells treated with the indicated concentrations of gemcitabine and 4 nucleosides without CVB3 infection were also analyzed for the cell viability by using the MTT assay.

Previously, we identified a new indication of gemcitabine as an effective anti-enteroviral inhibitor [15]. As a cytidine analog, gemcitabine is known to have an inhibitory activity on the pyrimidine biosynthesis. Besides, a few inhibitors of the pyrimidine biosynthesis have been reported to show the antiviral activity, at least partly, through activating the innate immune response [10, 11, 14]. Thus, in this study we sought to examine if the anti-enteroviral activity of gemcitabine is also associated with the modulation of pyrimidine biosynthesis and innate immunity. At first, in order to test if the anti-enteroviral activity of gemcitabine is related with the inhibition of pyrimidine biosynthesis, HeLa cells were infected with CVB3 and simultaneously treated with excessive 4 nucleosides (adenosine, guanosine, uridine and cytidine) in the presence of various doses of gemcitabine for 8 hours. Antiviral activity was measured by staining infected cells with an anti-CVB3 3Cpro antibody and secondary antibody conjugated with AF488 fluorescent dye and counting cells with a fluorescent signal. As previously reported, gemcitabine itself exhibited a strong antiviral activity on CVB3 infection, with an estimated IC_50_ of ∼5 μM and maximal efficacy of > 80 % (Figure 1A). When gemcitabine was co-treated with excessive 4 nucleosides each, only two pyrimidine nucleosides (cytidine and uridine) could significantly suppress the antiviral activity of gemcitabine, resulting in the sustained infection. Especially, cytidine had the strongest effect, which can be explained by the fact that gemcitabine is a cytidine analog. The effect of cytidine was evidently demonstrated by a dose-dependent suppression of gemcitabine’s antiviral activity (Supplementary Figure 1A). In contrast, purine nucleosides (adenosine and guanosine) had little effect. As a control experiment, cell viability was analyzed in the same condition by using MTT assay (Figure 1B). There were little changes except that the treatment with adenosine without gemcitabine decreased the cell viability by about 20 %, indicating the cytotoxic effect of adenosine. This phenomenon was further confirmed in an independent experiment using increasing concentrations of each nucleosides without CVB3 infection (Supplementary Figure 2).

To further confirm the effect of pyrimidine nucleosides on the antiviral activity of gemcitabine, CVB3 subgenomic replicon system was additionally used. This system contains the firefly luciferase gene in place of structural genes (VP4-VP1) of CVB3 viral genome and allows the quantitative measurement of viral replication [23, 24]. HeLa cells were transfected with *in vitro*-transcribed CVB3 replicon RNAs and simultaneously treated with 4 nucleosides each in the presence of various doses of gemcitabine for 8 hours. As similar to the results from CVB3 infection experiment (Figure 1A), addition of excessive pyrimidine nucleoside (cytidine or uridine) remarkably suppressed the antiviral activity of gemcitabine, thereby protecting the replication of CVB3 replicon (Supplementary Figure 3A). According to our previous study, gemcitabine has a strong antiviral effect on EV71 as well as CVB3 [15]. Therefore, we also tested pyrimidine nucleosides in EV71 replicon system. As a result, only cytidine and uridine suppressed the antiviral activity of gemcitabine on the replication of EV71 replicon, which is similar to those observed from CVB3-based assays (Supplementary Figure 3B). Collectively, these results strongly indicate the involvement of pyrimidine biosynthesis in the antiviral activity of gemcitabine.

### Suppression of the antiviral activity of gemcitabine by intermediates in the salvage pathway of pyrimidine biosynthesis

**Figure 2:**
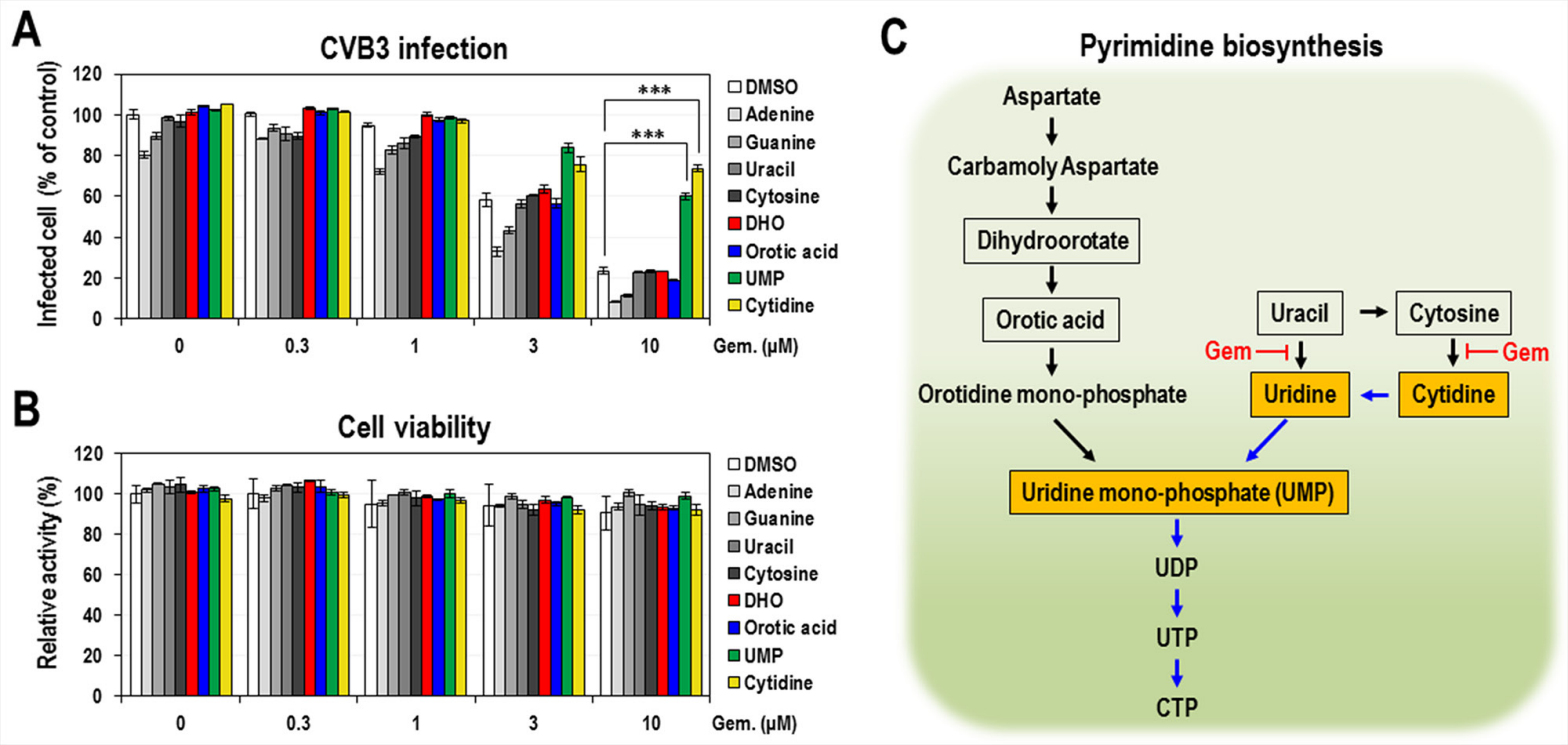
The effect of exogenous nucleobases and intermediates of pyrimidine biosynthetic pathway on the anti-CVB3 activity of gemcitabine Four nucleobases (adenine, guanine, cytosine and uracil) and intermediates (dihydroorotate, orotic acid and UMP) (100 μM) of pyrimidine biosynthetic pathway were treated as described in Figure 1A **(A)** and in Figure 1B **(B)**. Cytidine was used as a positive control. The average and standard deviation were obtained from three independent experiments. ^***^ indicates *P* < 0.001. **(C)** Pyrimidine biosynthetic pathway. Tested metabolites were indicated as boxes and metabolites with the significant effect were shown as orange boxes. The salvage pathway affected by gemcitabine was depicted by blue arrows.

The observation that the antiviral effect of gemcitabine is suppressed by the supplemented pyrimidine nucleosides directed us to further define the effect of gemcitabine on the pyrimidine biosynthetic pathway. Pyrimidine biosynthesis occurs by two separate pathways: *de novo* synthesis and the salvage (Figure 2C). To determine which pathway and step were affected by gemcitabine, a few intermediates of both pathways were tested. Dihydroorotate (DHO) and orotic acid were used as intermediates of *de novo* biosynthetic pathway, and 4 nucleobases (adenine, guanine, uracil and cytosine) were additionally used as intermediates of the salvage pathway. Uridine mono-phosphate (UMP) acting downstream of both pathways was also included. UMP is synthesized from uridine by uridine kinase in the salvage pathway and separately from orotidine mono-phosphate (OMP) by OMP decarboxylase in *de novo* biosynthetic pathway (Figure 2C). Gemcitabine, as a nucleoside analog, may directly affect the activity of enzyme(s) that are involved in the pyrimidine biosynthesis, resulting in the decrease of intermediates acting downstream of its target. Thus, excessive addition of any intermediate acting downstream of gemcitabine’s target can rescue the deficit and supposedly shows a suppressive effect on the antiviral activity of gemcitabine. When selected intermediates were co-treated with gemcitabine in CVB3-infected HeLa cells, only UMP significantly showed a suppressive effect with an extent similar to that of cytidine (Figure 2A). Other intermediates for *de novo* pathway (DHO and orotic acid) and for the salvage pathway (4 nucleobases) had marginal effects. Under the same condition, little changes in cell viability were shown (Figure 2B). Moreover, similar effect of UMP was also observed in CVB3 replicon-harboring cells (Supplementary Figure 4), even though it was slightly weaker than that of cytidine. Taken together the results from Figures 1 and 2, it seems that gemcitabine majorly targets the salvage pathway rather than *de novo* pathway, particularly uridine and/or cytidine synthesis from uracil and/or cytosine (Figure 2C).

### The activation of ISRE promoter by gemcitabine

**Figure 3:**
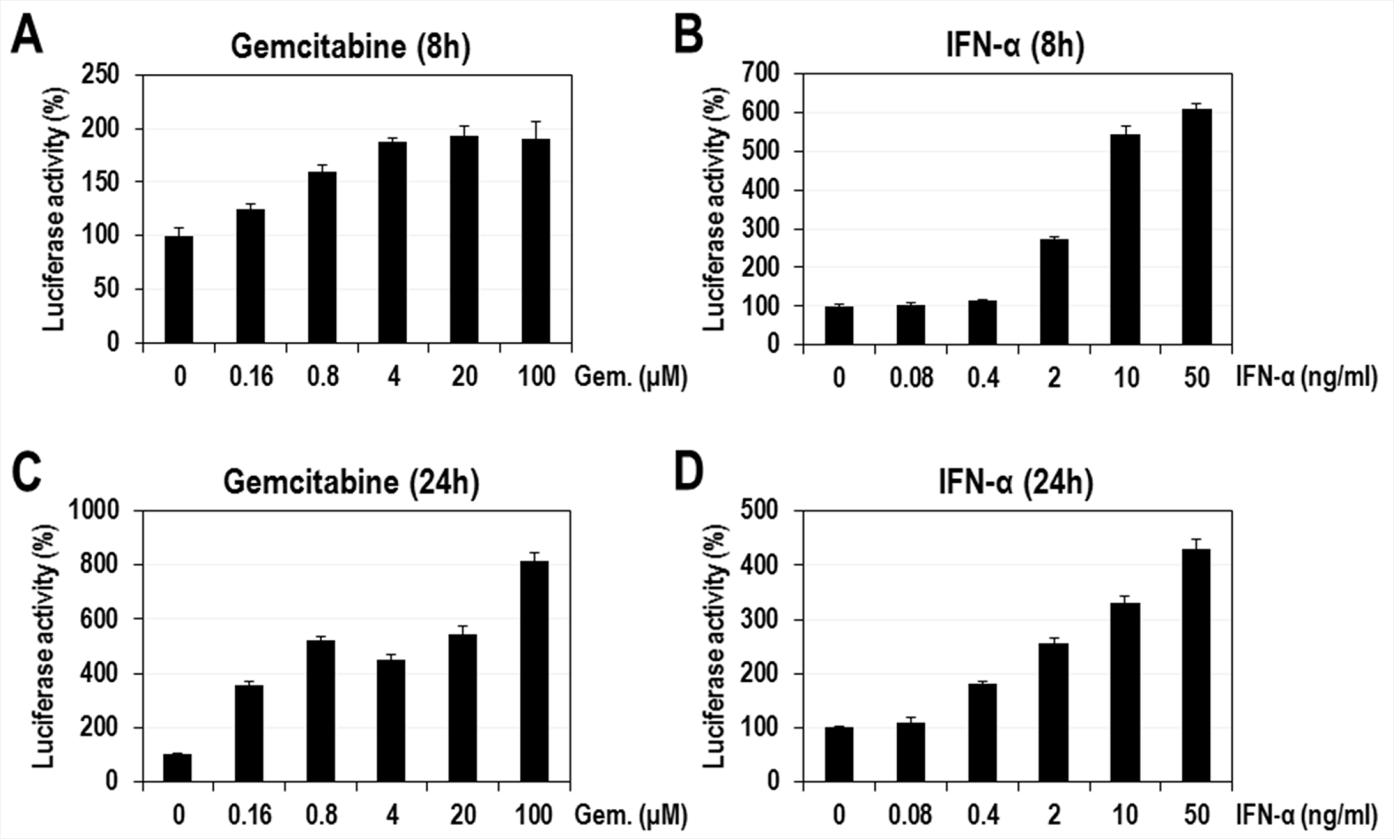
The effect of gemcitabine on ISRE promoter HeLa cells were transfected with plasmid encoding ISRE-luciferase and then treated with various concentrations of gemcitabine or IFN-α. At 8 or 24 hours after compound treatment cells were assayed for the firefly luciferase activity. **(A)** Gemcitabine (8h), **(B)** IFN-α (8h), **(C)** Gemcitabine (24h), and **(D)** IFN-α (24h). The relative luciferase activities in percentage were calculated by setting the value from DMSO-treated cells as 100%. The average and standard deviation were obtained from three independent experiments.

There are a few antiviral compounds interfering with the nucleoside biosynthesis, which subsequently induce innate immunity involving the upregulation of ISGs [11–13, 25]. In those studies antiviral compounds induced the expression of IFN-stimulated response element (ISRE)-luciferase reporter even without IFN stimulation. Thus, we also examined if gemcitabine itself has a stimulatory effect on ISRE-luciferase reporter. HeLa cells were transfected with the reporter plasmid and then treated with gemcitabine (0.16 to 100 μM) and IFN-α (0.08 to 50 ng/ml) for 8 or 24 hours. At 8 hours post-treatment gemcitabine increased the luciferase activity up to ∼ 200% of the control (Figure 3A). The increase of luciferase activity was much more evident at 24 hours post-treatment, maximally reaching ∼800% of the control (Figure 3C). In contrast, IFN-α induced the luciferase expression up to ∼600 and 400 % of the control at 8 and 24 hours post-treatment, respectively, indicating a rapid stimulation (Figure 3B and 3D). Both gemcitabine and IFN-α significantly stimulated the ISRE promoter but with apparently different time-kinetics. ISRE promoter more slowly responded to gemcitabine compared to IFN-α. Differential mechanisms of ISRE activation by gemcitabine and IFN-α will be proposed in the discussion section.

To further confirm that ISRE activation by gemcitabine is correlated with its antiviral activity, we tested if the antiviral activity is enhanced by the longer treatment of gemcitabine. HeLa cells were treated with gemcitabine for 16 hours prior to CVB3 infection and maintained for another 8 hours, achieving 24 hours of treatment (24h exposure). Antiviral activity was assessed by quantifying the virus-infected cells showing a fluorescent signal of 3Cpro protein at 24 hours after treatment with gemcitabine. As a result, a considerably stronger antiviral effect was exhibited by a longer time treatment (24h exposure) than a shorter time treatment (8h exposure) (Supplementary Figure 5). The estimated IC_50_ of gemcitabine in ‘24h exposure’ cells (∼0.2 μM) was much lower than that in ‘8h exposure’ cells (∼1 μM). Moreover, the maximum efficacy of gemcitabine in ‘24h exposure’ cells (∼100%) was apparently higher than that in ‘8h exposure’ cells (∼80%). These results indicate that the enhanced antiviral activity by a longer exposure of cells with gemcitabine is likely correlated with stronger activation of ISRE promoter attained at 24 hours of treatment.

### The activation of ISGs by gemcitabine through the inhibition of pyrimidine biosynthesis

**Figure 4:**
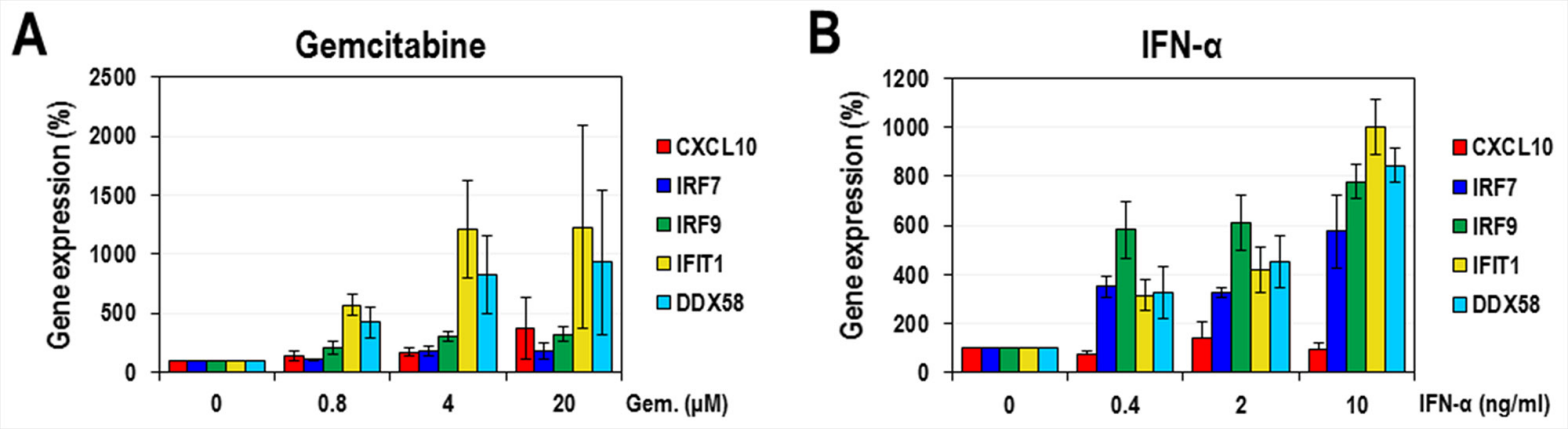
The effect of gemcitabine on ISGs HeLa cells were treated with various concentrations of gemcitabine **(A)** or IFN-α **(B)**. After 24 hours, cellular RNAs were isolated and the expression levels of 5 ISGs (CXCL10, IRF7, IRF9, IFIT1 and DDX58) were quantified using real-time PCR. Results were normalized as a percentage relative to DMSO-treated cells. The average and standard deviation were obtained from three independent experiments.

**Figure 5:**
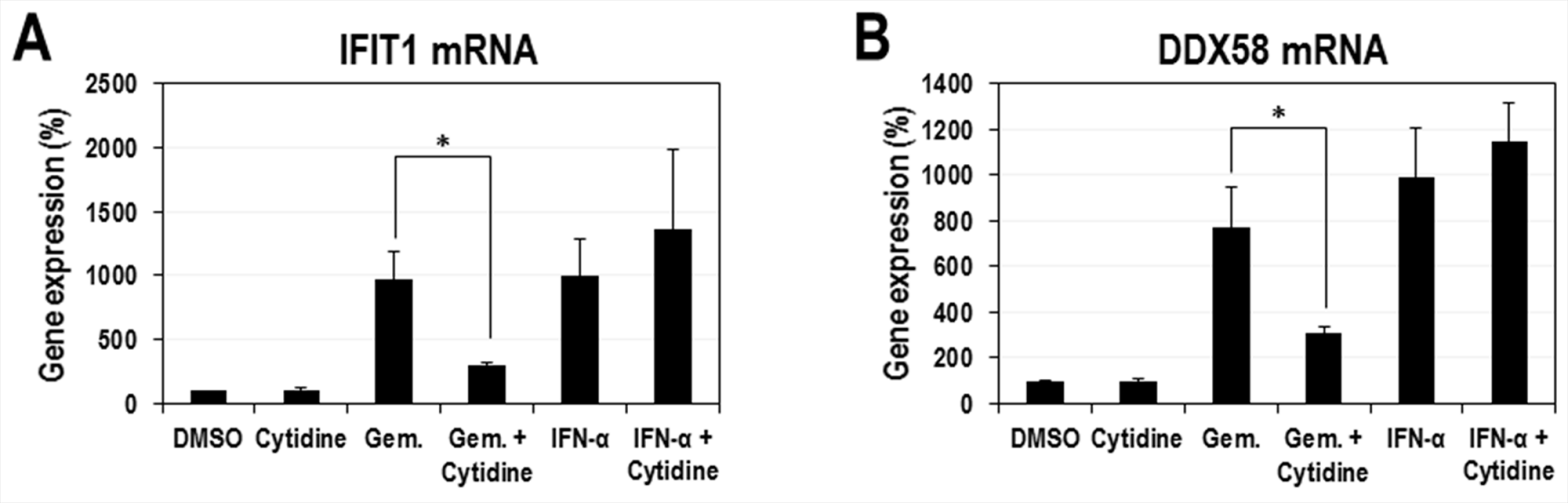
Suppression of gemcitabine-induced ISG expression by exogenous cytidine HeLa cells were treated with gemcitabine (20 μM) or IFN-α (10 ng/ml) in the presence or absence of cytidine (50 μM). After 24 hours, cellular RNAs were isolated and the expression of IFIT1 **(A)** and DDX58 **(B)** mRNAs was determined by quantitative real-time PCR. Results were normalized as a percentage relative to DMSO-treated cells. The average and standard deviation were obtained from three independent experiments. ^*^ indicates *P* < 0.05.

IFNs trigger an intracellular signal through the JAK/STAT pathway and transcriptionally induce numerous ISGs under the control of ISRE [26]. According to our aforementioned results, gemcitabine itself has a stimulatory effect on ISRE promoter like IFNs. However, their modes of activation seem to be quite different. Thus, in order to further confirm the activation of ISRE-containing promoters by gemcitabine and to know how differently these promoters respond to gemcitabine and IFN-α, the transcriptional expression of 5 ISGs (CXCL10, IRF7, IRF9, IFIT1, and DDX58) were analyzed. HeLa cells were treated with increasing doses of gemcitabine or IFN-α for 24 hours and then total RNAs were prepared for quantitative real-time PCRs. As a result, gemcitabine strongly induced two genes (IFIT1 and DDX58) up to more than 7-fold of the control and moderately three genes (CXCL10, IRF7 and IRF9) up to less than 4-fold of the control (Figure 4A). On the other hand, IFN-α strongly induced 4 ISGs (IRF7, IRF9, IFIT1 and DDX58) up to more than 5-fold of the control (Figure 4B). These results indicate that gemcitabine has a different mode of ISG activation from IFN-α. In order to further confirm whether the ISG activation by gemcitabine is mediated by the modulation of pyrimidine biosynthesis, we tested the effect of cytidine on the activation of IFIT1 and DDX58 genes, the two strongest responders to gemcitabine. As expected, the supplemented cytidine considerably suppressed the gemcitabine-induced expression of IFIT1 and DDX58 (Figure 5). In contrast, cytidine had no significant effect on the IFN-induced IFIT1 and DDX58 expression.

To further clarify whether the stimulation of innate immune response by gemcitabine is independent of IFN-induced JAK/STAT pathway, we first examined the phosphorylation of STAT1 at Tyr 701, a major event occurred at the early step of IFN signaling. HeLa cells were treated with various doses of gemcitabine or IFN-α for 2 hours or 24 hours and then cell lysates were analyzed by Western blotting. As a result, the levels of phosphorylated STAT1 (pTyr701) proteins was not changed at all by gemcitabine at both 2 and 24 hours of treatment (Supplementary Figure 7A and 7C). On the contrary, treatment with IFN-α remarkably increased the phosphorylation of STAT1 protein at both time conditions (Supplementary Figure 7B and 7D), as expected. In addition, we further examined whether the gemcitabine-induced ISG expression involved IRF9, which forms IFN-stimulated gene factor 3 (ISGF3) complex by interacting with the phosphorylated STAT1/STAT2 and functions as a transcriptional activator of ISGs [27]. siRNA-mediated knockdown of IRF9 did not affect the gemcitabine-induced expression of DDX58, while it had a significant reducing effect on that induced by IFN-α (decreased by ∼45% of the control) (Supplementary Figure 8).

Collectively, these results demonstrate that the antiviral effect of gemcitabine involves the activation of ISGs, the mode of which is different from IFN-dependent conventional way but which is mediated, at least partly, by the inhibition of pyrimidine biosynthesis

## DISCUSSION

Gemcitabine, currently in use for cancer therapy, has the antiviral activity against a broad range of RNA viruses, including poliovirus [19], HCV [16], influenza A [18], HIV [17], MERS-CoV [20] and ZIKV [21]. In the previous study, we also identified gemcitabine as an effective and potent anti-enteroviral agent [15]. Herein, we further defined the underlying mechanism of gemcitabine’s antiviral action that involved the modulation of pyrimidine biosynthesis and the subsequent activation of ISGs.

There have been accumulating reports that nucleoside analogs act as the antiviral agents through the modulation of nucleotide biosynthesis [10, 28–30]. As a cytidine analog, gemcitabine was also expected to work in this way. Indeed, the antiviral activity of gemcitabine in both CVB3-infected and CVB3 replicon-harboring HeLa cells was significantly suppressed by the addition of exogenous pyrimidine nucleosides (cytidine and uridine) but not by purine nucleosides (Figure 1 and Supplementary Figure 3). Further delineation demonstrated that gemcitabine acted through the salvage pathway rather than *de novo* pathway and targeted most probably uridine and/or cytidine synthesis from uracil and/or cytosine (Figure 2 and Supplementary Figure 4). Therefore, it is conceivable that gemcitabine induces the decrease of pyrimidine nucleosides and their subsequent metabolites including UMP.

There have been a few reports that the inhibition of pyrimidine biosynthesis induces the innate immune response, especially the activation of ISGs, which is the major antiviral mechanism in the early stage of virus infection [10, 11, 14]. These previous findings were confirmed in our test by observing the activation of ISRE promoter by leflunomide, an inhibitor of dihydroorotate dehydrogenase (DHODH) on the pyrimidine biosynthetic pathway (Supplementary Figure 6). More importantly, we newly revealed that gemcitabine also activated the ISRE promoter (Figure 3A and 3C). The activation of ISRE promoter by gemcitabine was further confirmed by the significant induction of all 5 endogenous ISGs (CXCL10, IRF7, IRF9, IFIT1, and DDX58) tested, which was evaluated by quantitative real-time PCR analysis (Figure 4A). Intriguingly, the increase of IFIT1 and DDX58 mRNAs was particularly outstanding, and the increase of those mRNAs was definitely suppressed by the additional treatment with cytidine, suggesting the role of IFIT1 and DDX58 in the antiviral effect of gemcitabine. IFIT1 family members are among the most highly induced ISGs in response to IFN [31] and there are many reports concerning their antiviral functions on various viruses. For instance, IFIT1 proteins exhibit the antiviral effect generally by sequestrating viral genomes having 5′-triphosphates [32]. They also inhibit HCV translation initiation by binding to eIF3, which is required for the efficient ribosome recruitment on HCV IRES [33, 34]. In addition, IFIT’s targeting of HPV E1 helicase, which is essential for viral DNA replication [35], was reported as anti-Human Papillomavirus (HPV) effect of IFIT protein. DDX58, the other ISG strongly stimulated by gemcitabine, encodes retinoic acid-inducible gene I (RIG-I). It is part of the RIG-I-like receptor family, which functions as a pattern recognition receptor that is a sensor for single- or double-strand RNA of viruses such as flavivirus, influenza A and reovirus, and is involved in triggering an antiviral response [36–38]. Also, IFIT and DDX58 proteins may majorly contribute to the antiviral action of gemcitabine in a way similar to the aforementioned mechanisms. Still, we would not exclude the contribution of other ISGs such as CXCL10, IRF7, and IRF9, and other untested ISGs as effectors to the full antiviral activity of gemcitabine.

One of the most interesting observations from our study is that gemcitabine seems to activate the expression of ISGs in a way different from IFN-α. First, ISRE promoter more slowly responded to gemcitabine, compared to IFN-α (Figure 3). Gemcitabine weakly activated the ISRE promoter at 8 hours and almost fully at 24 hours after treatment, which is quite contrary to IFN-α that achieved the full activation even at 8 hours after treatment. Second, the pattern of ISG activation was quite different between gemcitabine and IFN-α (Figure 4). Gemcitabine exhibited a relatively strong induction of IFIT1 and DDX58 mRNAs over IRF7 and IRF9 mRNAs, while IFN-α had a similarly strong stimulatory effect on all of IRF7, IRF9, IFIT1 and DDX58 mRNAs. Considering that all 4 ISGs contain STAT1/2-binding element in their promoters, the mode of gemcitabine seems to be weakly dependent on STAT1/2 or independent of them, which is contrary to IFN-α that highly depends on them. Third, the phosphorylation of STAT1 (Tyr 701) was not induced by gemcitabine, while a remarkable induction of STAT1 phosphorylation was observed by IFN-α (Supplementary Figure 7). Fourth, the expression of DDX58 mRNAs induced by gemcitabine was not affected by siRNA-mediated IRF9 knockdown, which is contrary to the result that the IFN-α-induced expression of DDX58 mRNAs was significantly reduced at the same condition (Supplementary Figure 8). Fifth, the gemcitabine-induced expression of IFIT1 and DDX58 mRNAs was significantly suppressed by the additional treatment with cytidine, while that by IFN-α was not affected (Figure 5). This observation is of particular significance in that it strongly suggests a crosstalk between the pyrimidine biosynthesis and the innate immune response, which is induced by gemcitabine but not by IFN-α. Decreased pyrimidine pool or inactivation of metabolic enzyme(s) might trigger a signal, which is delivered to certain *cis*-acting element on a subset of ISGs possibly through the relay of some kinase(s). As mentioned above, this signal is less likely to be dependent on STAT1/2-IRF9 (IFN-stimulated gene factor 3; ISGF3) that is the major transcriptional complex in the IFN-induced JAK/STAT pathway [27]. Rather, the possible role of IRF3 or IRF7 needs to be alternatively evaluated. Further investigation in the following study will clarify the underlying molecular mechanism of gemcitabine’s antiviral action.

Our results, particularly the suppression of the gemcitabine-induced ISG activation by cytidine (Figure 5), indicate that the gemcitabine-induced innate immune response is mediated, at least partly, by its pyrimidine-depleting effect. Moreover, according to the results in Figure 1A and 2A, the gemcitabine’s pyrimidine-depleting effect seems to occur mainly through its targeting of the salvage pathway. However, considering that neither excessive cytidine, uridine nor UMP could achieve the full suppression of gemcitabine’s antiviral effect but by up to 50-70% of DMSO control (Figure 1A and 2A), there seems to be the other way of contribution that accounts for the residual portion of antiviral effect (∼30-50%). Besides the salvage pathway of pyrimidine biosynthesis, gemcitabine is known to inhibit ribonucleotide reductase catalyzing the formation of dNTPs from NTPs [39], which is another cause of nucleotide depletion. Thus, the residual antiviral activity might be explained by these additional inhibition of nucleotide biosynthesis, seemingly resulting in the severe nucleotide depletion and the subsequent stronger ISG activation. Alternatively, gemcitabine could be incorporated into newly synthesized viral genome or directly inhibit RDRP activity, resulting in the reduced viral replication.

In this study, we demonstrated the involvement of pyrimidine inhibition-induced innate immune response in the antiviral activity of gemcitabine, particularly by targeting the salvage pathway. The antiviral action of gemcitabine through innate immunity supported the feasibility of its therapeutic application as a broad-spectrum antiviral drug.

## MATERIALS AND METHODS

### Cells, viruses, antibodies and other reagents

HeLa cells were purchased from ATCC and cultured as previously described [15]. The stock of CVB3 (Nancy; ATCC VR-30) was also purchased from ATCC and amplified in HeLa cells. Gemcitabine, nucleosides (adenosine, guanosine, cytidine and uridine), nucleobases (adenine, guanine, cytosine and uracil), pyrimidine synthetic intermediates [dihydroorotate (DHO), orotic acid and uridine mono-phosphate (UMP)], IFN-α-2A and leflunomide were purchased from Sigma-Aldrich. Rabbit polyclonal anti-CVB3 3Cpro antibody was generated in house by immunizing with CVB3 3Cpro recombinant protein. Anti-rabbit secondary antibody conjugated with Alexa Fluor 488 (AF488) was purchased from Life technologies.

### Antiviral activity assay

To examine the involvement of pyrimidine biosynthetic pathway in the antiviral activity of gemcitabine, HeLa cells were infected with CVB3 (5 MOI) and simultaneously treated with nucleosides, nucleobases or pyrimidine biosynthetic intermediates (100 μM). At 8 hours post-infection cells were fixed with ice-cold mixture of methanol-acetone (3:1, *v*/*v*). After washing twice with PBS, infected cells were stained with rabbit polyclonal anti-CVB3 3Cpro antibody and anti-rabbit secondary antibody conjugated with AF488. Nuclei were counterstained with Hoechst 33342 (Life technologies). Images were captured and viral infection was quantified as previously described [15]. Infection was calculated as a percentage relative to DMSO control.

### Cell viability assay

MTT assay was used for measuring cell viability as described previously [40]. Cell viability was calculated as a percentage relative to the control.

### Replicon assay

Plasmids p53CB3-LUC and pRibFluc-EV71, which contain the firefly luciferase gene in place of the P1 capsid coding region of CVB3 and EV71 viral genomes, were kindly provided by Frank J. M. van Kuppeveld (Utrecht University, The Netherlands) [23, 24]. Synthesis of CVB3 and EV71 replicon RNAs and replicon assays were performed as previously described [40]. Briefly, HeLa cells (3×10^5^cells/well) on a 6-well plate were transfected with 0.4 μg of replicon RNA, split into 96-well plates (2×10^4^ cells/well), and simultaneously treated with nucleosides, nucleobases or pyrimidine biosynthetic intermediates in the presence of gemcitabine. Eight hours after compound treatment, cells were assayed for the firefly luciferase activity using One-Glo Luciferase Assay system (Promega).

### ISRE-luciferase reporter assay

HeLa cells (3×10^5^cells/well) in a 6-well plate were transfected with an ISRE-luciferase reporter plasmid using Lipofectamine 2000 (Invitrogen) and split into 96-well plates (2 × 10^4^ cells/well). The IFN-stimulated response elements (ISRE)-luciferase reporter plasmid was described previously [41]. Forty-eight hours after transfection, cells were treated with gemcitabine or IFN-α for another 8 or 24 hours. Thereafter, cells were assayed for the firefly luciferase activity using One-Glo Luciferase Assay System (Promega).

### Quantitative real-time PCR of ISGs

Total RNAs from cells treated with compounds in a 6-well plate were prepared using RNeasy mini kit (QIAGEN) and then cDNAs were generated using SuperScript IV (Life Technologies) and random hexamers. For the quantification of ISG expression, we performed a real-time PCR using SsoFast Evagreen Supermix (Bio-Rad) and primers described in the previous report [10]. The β-actin mRNA was also quantified and used as the endogenous control [42].

### Western blotting

HeLa cells (3×10^5^cells/well) in a 6-well plate were treated with gemcitabine or IFN-α for 2 or 24 hours. Cell lysates were prepared and subjected to Western blot analysis with anti-STAT1 and -phospho-STAT1 (Tyr 701) antibodies. GAPDH proteins were also analyzed as a loading control. More detailed procedures were described previously [43]. Anti-STAT1 and -phospho-STAT1 (Tyr 701) antibodies were purchased from Cell Signaling Technology, and GAPDH antibody was purchased from Santa Cruz Biotechnology.

### siRNA-mediated knockdown of IRF9

HeLa cells (3×10^5^cells/well) in a 6-well plate were transfected with IRF9 or negative control siRNAs by using Lipofectamine RNAiMAX reagent (Invitrogen) for 24 hours and then treated with gemcitabine or IFN-α for another 24 hours. Thereafter, total RNAs were prepared and analyzed for the quantitative real-time PCR of DDX58 mRNAs. The siRNAs were synthesized by BIONEER (Daejeon, South Korea).

### Statistical analysis

In figures, data are presented as the means ± standard deviations obtained from 3 or more independent experiments. ^*^, ^**^, and ^***^ represent p<0.05, p<0.01, and p<0.001, respectively, which were analyzed by Student’s *t-*test.

## SUPPLEMENTARY MATERIALS FIGURES


