# Supplementary material for: Gemcitabine, a broad-spectrum antiviral drug, suppresses enterovirus infections through innate immunity induced by the inhibition of pyrimidine biosynthesis and nucleotide depletion
Source: Oncotarget. 2017 Dec 15;8(70):115315–25. doi: 10.18632/oncotarget.23258 (PMC5777774; doi:10.18632/oncotarget.23258)
Supplement: Supplementary file 1 [file oncotarget-08-115315-s001.pdf]

Gemcitabine, a broad-spectrum antiviral drug, suppresses enterovirus infections through innate immunity induced by the inhibition of pyrimidine biosynthesis and nucleotide depletion

SUPPLEMENTARY MATERIALS

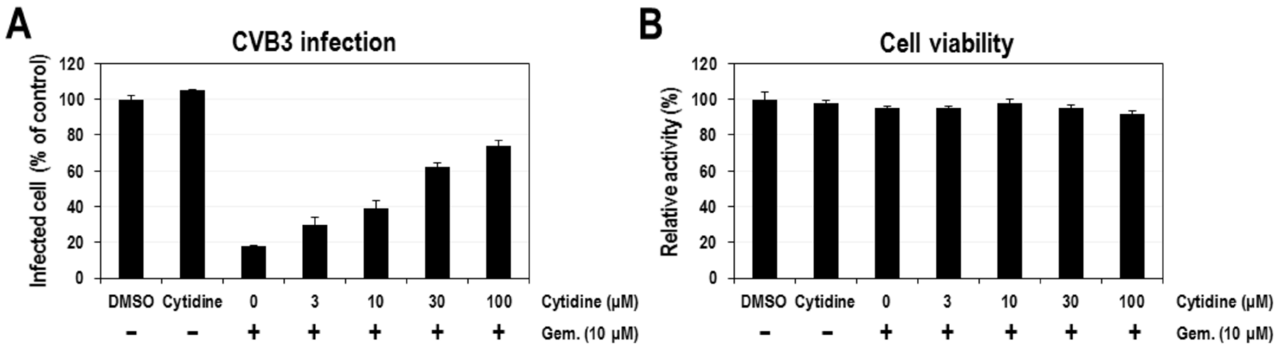

Supplementary Figure 1: Dose-dependent suppression of gemcitabine-induced anti-CVB3 effect by cytidine. **(A)** HeLa cells were infected with CVB3 and immediately treated with increasing concentrations of cytidine and 10 µM of gemcitabine. At 8 hours post-infection the virus-infected cells were visualized by staining with anti-CVB3 3Cpro antibody, and the percentage of infected cells among total cells was calculated by setting the value from DMSO-treated cells as 100%. **(B)** HeLa cells treated with indicated doses of cytidine and gemcitabine without CVB3 infection were also analyzed for the cell viability by using MTT assay. The average and standard deviation were obtained from three independent experiments.

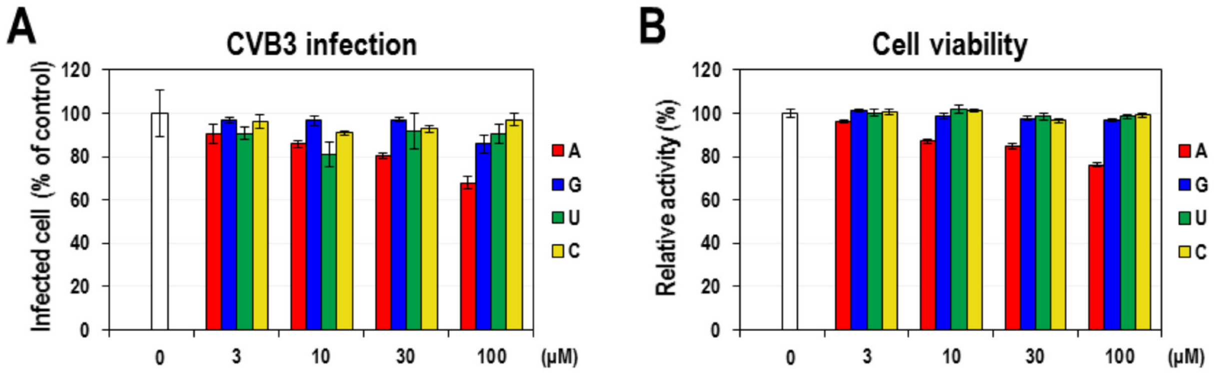

Supplementary Figure 2: **Little anti-CVB3 activity of nucleosides** **(A)** HeLa cells were infected with CVB3 and simultaneously treated with the indicated concentrations of 4 nucleosides. At 8 hours post-infection the virus-infected cells were visualized by staining with anti-CVB3 3Cpro antibody, and the percentage of infected cells among total cells was calculated by setting the value from DMSO-treated cells as 100%. **(B)** HeLa cells treated with indicated doses of 4 nucleosides without CVB3 infection were also analyzed for the cell viability by using MTT assay. The average and standard deviation were obtained from three independent experiments.

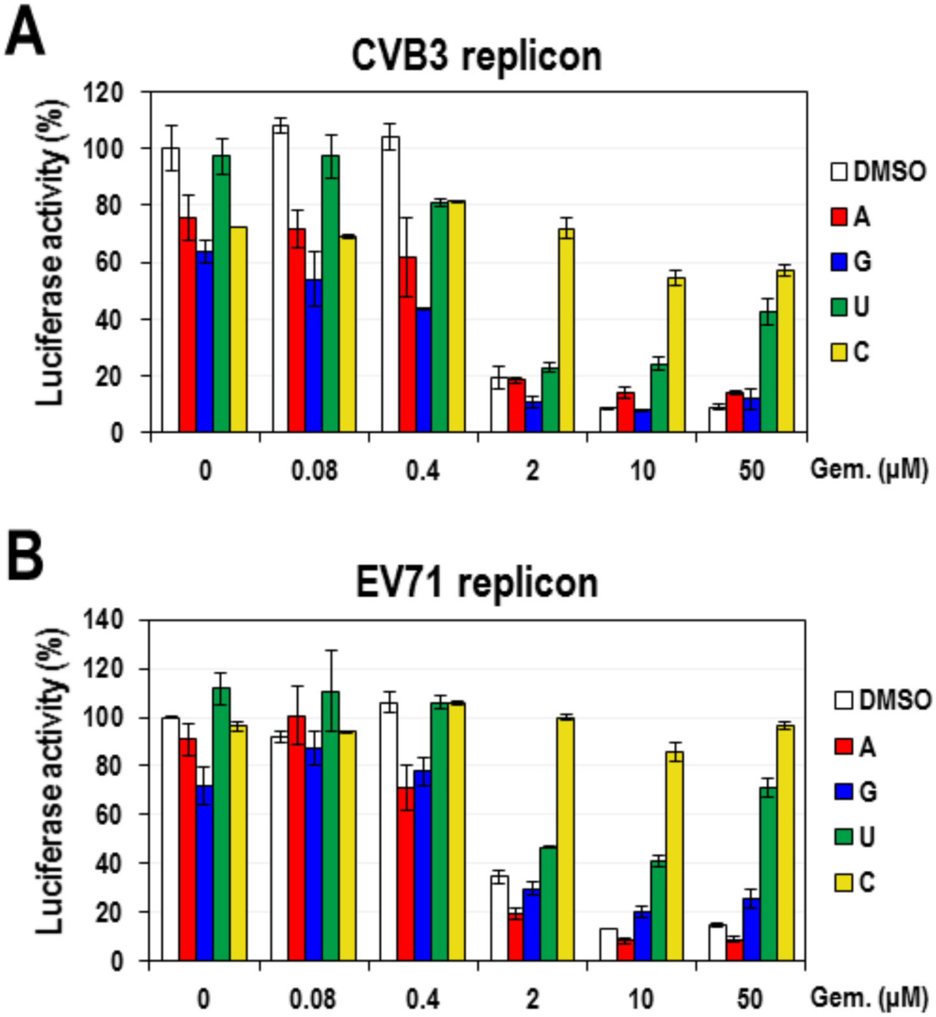

Supplementary Figure 3: Suppression of anti-enteroviral effect of gemcitabine by exogenous pyrimidine nucleosides. HeLa cells were transfected with *in vitro*-transcribed CVB3 **(A)** or EV71 **(B)** replicon RNAs, simultaneously treated with the indicated concentrations of gemcitabine and 100 μM of 4 nucleosides for 8 hours, and then assay for the firefly luciferase activity. The relative luciferase activities in percentage were calculated by setting the value from DMSO-treated cells as 100%. The average and standard deviation were obtained from three independent experiments.

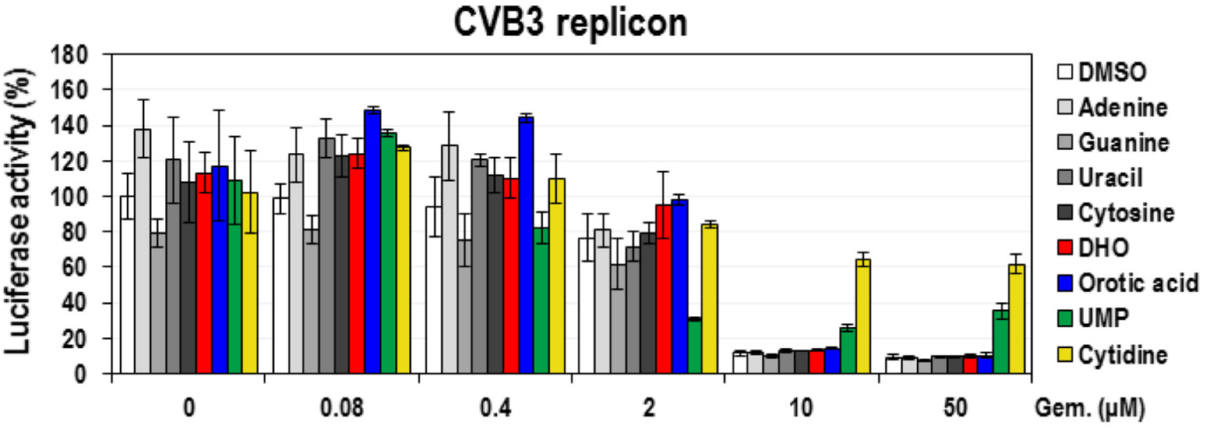

Supplementary Figure 4: The effect of exogenous nucleobases and intermediates of pyrimidine biosynthetic pathway on the anti-CVB3 activity of gemcitabine. Four nucleobases (adenine, guanine, cytosine and uracil) and intermediates (dihydroorotate, orotic acid and UMP) (100 μM) of pyrimidine biosynthetic pathway were treated as described in Supplementary Figure 3A. Cytidine was used as a positive control. The average and standard deviation were obtained from three independent experiments.

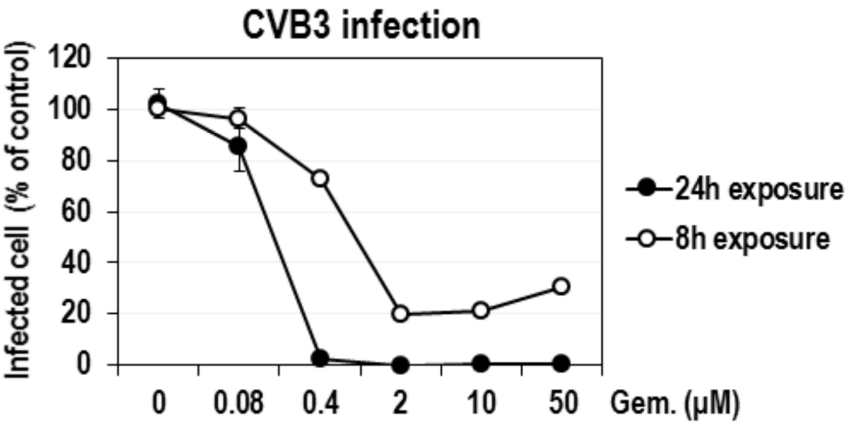

Supplementary Figure 5: Antiviral activity enhanced by the longer treatment with gemcitabine. HeLa cells were pre-treated with gemcitabine for 16 hours and then infected with CVB3 for another 8 hours (24h exposure in total). Thereafter, the virus-infected cells were visualized by staining with anti-CVB3 3Cpro antibody, and the percentage of infected cells among total cells was calculated by setting the value from DMSO-treated cells as 100%. HeLa cells treated with gemcitabine, simultaneously infected with CVB3, and then incubated just for 8 hours (8h exposure), were analyzed for the comparison. The average and standard deviation were obtained from three independent experiments.

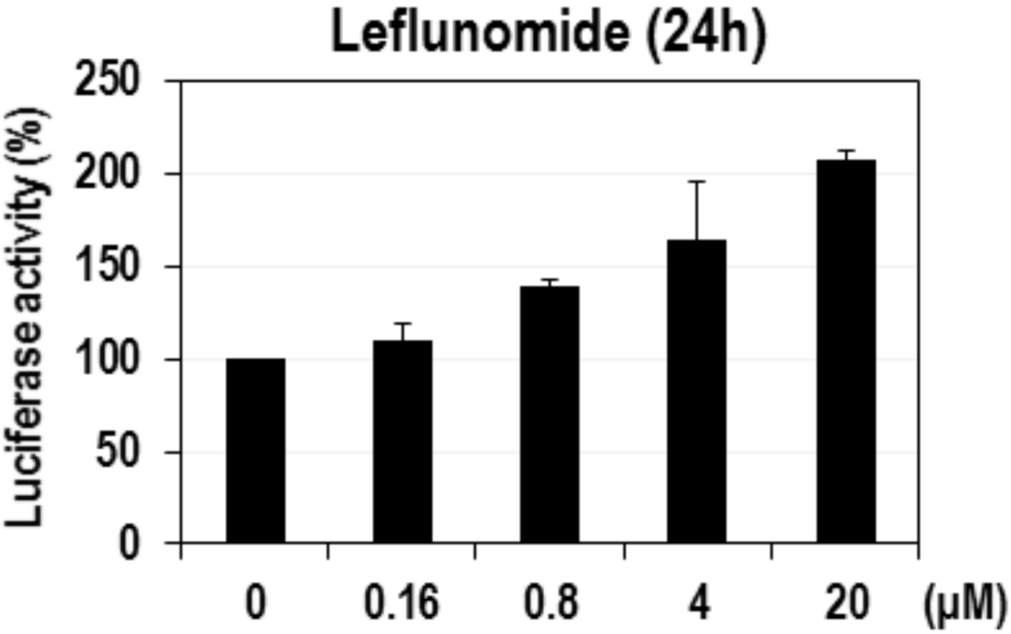

Supplementary Figure 6: The effect of leflunomide on ISRE promoter. HeLa cells were transfected with plasmid encoding ISRE-luciferase and then treated with the indicated concentrations of leflunomide for 24 hours and assayed for the firefly luciferase activity. The average and standard deviation were obtained from three independent experiments.

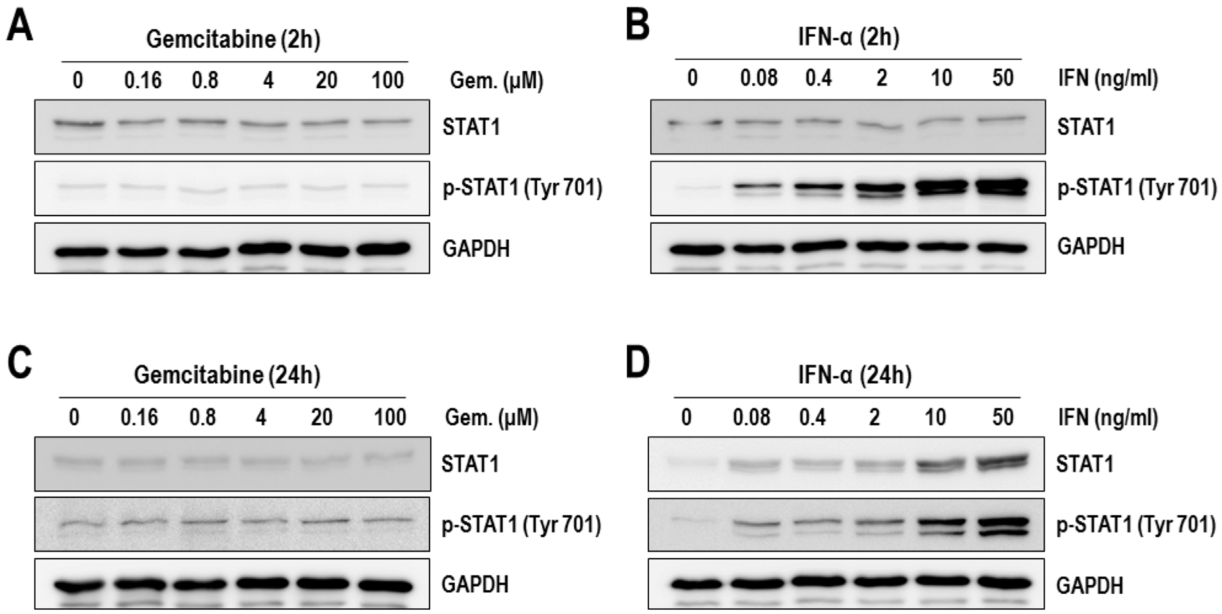

Supplementary Figure 7: The effect of gemcitabine on the phosphorylation of STAT1 at Tyr701. HeLa cells were treated with gemcitabine or IFN-α for 2 hours or 24 hours. **(A)** Gemcitabine (2h), **(B)** IFN-α (2h), **(C)** Gemcitabine (24h), and **(D)** IFN-α (24h). Total cell lysates were harvested and subjected to Western blot analysis against STAT1 and phospho-STAT1 (Tyr 701). GAPDH protein was also analyzed as a loading control.

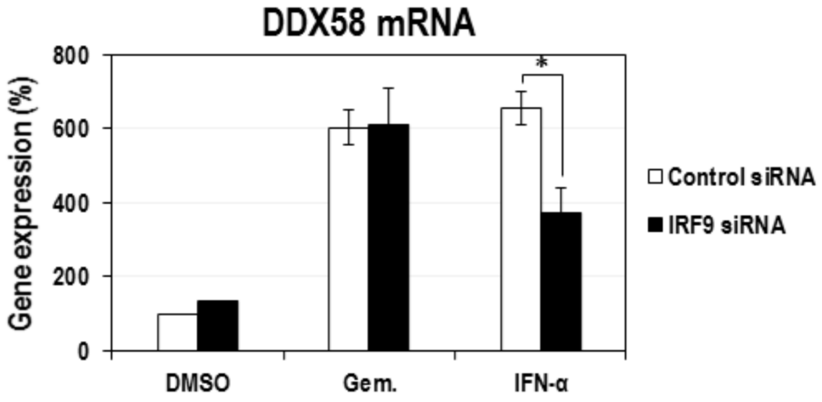

Supplementary Figure 8: The effect of IRF9 knockdown on the expression of ISGs induced by gemcitabine or IFN-α. HeLa cells were transfected with IRF9 or negative control siRNAs and then treated with gemcitabine (20 μM) or IFN-α (10 ng/ml). After 24 hours, total RNAs were prepared and the level of DDX58 mRNAs was quantified and presented as described in the legend of Figure 4. The average and standard deviation were obtained from three independent experiments. * indicates $P < 0.05$.
